# Oral health-related quality of life in Colombian Older Adults

**DOI:** 10.1093/geroni/igab046.2273

**Published:** 2021-12-17

**Authors:** Margarita Osuna, Piedad Suarez, Jennifer Ailshire

**Affiliations:** 1 USC, University of Southern California, California, United States; 2 University of Southern California, University of Southern California, California, United States; 3 University of Southern California, Los Angeles, California, United States

## Abstract

Despite its importance for health and wellbeing, oral health quality of life (OHQoL) has received little attention in lower-income countries, such as Colombia. This study describes the prevalence of older adults’ OHQoL and variability by socioeconomic status. We use data from the 2015 SABE-Colombia (N=18,700), a nationally representative survey of community-dwelling Colombians ages 60 and older. We used the Simple Count Geriatric Oral Health Assessment Index (SC-GOHAI), a self-reported measure of frequent oral health problems such as chewing, swallowing, and speaking designed to assess OHQoL. The scale ranges from 0-12; higher scores indicate worse OHQoL. About 69% of older Colombians reported at least one OHQoL problem. The most common issues were difficulty chewing hard food and speaking. High education and income were associated with better OHQoL and smoking were associated with worse OHQoL. Oral health may therefore reflect another dimension of social and health inequality for older Colombians.

